# Exploring the value of new preoperative inflammation prognostic score: white blood cell to hemoglobin for gastric adenocarcinoma patients

**DOI:** 10.1186/s12885-019-6213-0

**Published:** 2019-11-21

**Authors:** Hua-Long Zheng, Jun Lu, Jian-Wei Xie, Jia-Bin Wang, Jian-Xian Lin, Qi-Yue Chen, Long-Long Cao, Mi Lin, Ru-Hong Tu, Ze-Ning Huang, Ju-Li Lin, Ping Li, Chao-Hui Zheng, Chang-Ming Huang

**Affiliations:** 1 Department of Gastric Surgery, Fujian Medical University Union Hospital, No.29 Xinquan Road, Fuzhou, 350001 Fujian Province China; 2 Department of General Surgery, Fujian Medical University Union Hospital, Fuzhou, Fujian Province China; 3 Key Laboratory of Ministry of Education of Gastrointestinal Cancer, Fujian Medical University, Fuzhou, Fujian Province China; 4 Fujian Key Laboratory of Tumor Microbiology, Fujian Medical University, Fuzhou, Fujian Province China

**Keywords:** Gastric cancer, Preoperative inflammation-based prognostic scores (PIPS), Long-term survival

## Abstract

**Background:**

The platelet to lymphocyte ratio (PLR), lymphocyte to monocyte ratio (LMR), and neutrophil to lymphocyte ratio (NLR) reflect the systematic inflammatory response, with some evidence revealing that they are associated with poorer survival in patients with gastric cancer. However, the effect of the white blood cell to hemoglobin ratio (WHR) on the long-term prognosis of patients with gastric cancer has not been reported. Therefore, we sought to characterize the effect of WHR on long-term survival after radical gastrectomy and compare its value with that of other preoperative inflammation-based prognostic scores (PIPS).

**Methods:**

Data from 924 patients with a diagnosis of nonmetastatic gastric adenocarcinoma who underwent surgical resection between December 2009 and May 2013 were included in this study.

**Results:**

The optimal cutoff values for the WHR, PLR, LMR, and NLR were 2.855, 133.03, 3.405, and 2.61, respectively. Patients with an increased WHR (53% vs. 88.1%, *p* < 0.001), PLR (60.9% vs 75.6%, *p* < 0.001) and NLR (56.7% vs 72.8%, *p* < 0.001) and a decreased LMR (54% vs 74.5%, *p* < 0.001) had a significantly decreased 5-year OS. However, the stratified analysis showed that only the WHR predicted a significant 5-year survival rate difference at each stage as follows: stage I (82.7% vs 94.3%, *p* = 0.005), stage II (71.3% vs 90.2%, *p* = 0.001) and stage III (38.2% vs 58.1%, *p* < 0.001). The time-ROC curve showed that the predictive value of the WHR was superior to that of the PLR, LMR, and NLR during follow-up. The WHR (0.624) C-index was significantly greater than the PLR (0.569), LMR (0.584), and NLR C-indexes (0.56) (all *P* < 0.001).

**Conclusion:**

Compared with other PIPS, the WHR had the most powerful predictive ability when used for the prognosis of patients with gastric adenocarcinoma.

## Background

Although the incidence and mortality rates of gastric cancer are decreasing, approximately 951,600 new cases of gastric cancer were diagnosed, and 723,100 people died of gastric cancer in 2012. In general, Eastern Asia, including Korea, Japan, and China, has the greatest incidence rate [1]. Clearly, there is a genetic basis for cancer development, and recent research suggests that host inflammatory responses play an important role in cancer development and disease progression [2, 3]. The recognition of cancer inflammation as an important hallmark of cancer [3], as well as the recognition of the important role of the immune system in cancer surveillance and elimination [4], have led to the examination of various inflammatory markers as prognostic factors for cancer [5]. Many inflammation-based scores, for example, the neutrophil to lymphocyte ratio (NLR), platelet to lymphocyte ratio (PLR), and lymphocyte to monocyte ratio (LMR), have all been compared and attracted interest because of their prognostic value for gastric cancer due to the fact that their calculation is easy to perform for all patients undergoing radical gastrectomy at no extra cost [6–14].

Among these scores, the NLR was the most widely used for a variety of malignancies. A number of studies have shown that the elevation of NLR is associated with a worse survival rate for patients with malignant tumors, including colorectal cancer [15], pancreatic cancer [16], gastrointestinal stromal tumor [17], hepatocellular carcinoma [18], non–small cell lung cancer [19], ovarian cancer [20], multiple myeloma [21], renal cell carcinoma [22], and gastric cancer [6–8]. Zhang, Y. and Lu et al. reported that elevated PLR was related to poor prognosis in gastric cancer patients before treatment [9–11]. Recently, many studies have demonstrated the association between pretreatment LMR and prognosis in patients with gastric cancer and concluded that LMR could reflect the extent of systemic inflammation [12–14]. However, most of the orevious studies focused on specific blood cells. In our opinion, as the most important part of the immune system, WBCs should be considered an integral cell type, and to the best of our knowledge, no one has investigated the white blood cell to hemoglobin ratio (WHR). Therefore, the purpose of this study was to explore the value of the WHR, to compare the values of different inflammation scores that are derived from a full blood count and to select the best score for gastric cancer patients.

## Methods

A prospectively maintained database was reviewed to analyze all patients who were diagnosed with gastric cancer and who underwent potentially radical gastrectomy between December 2009 and May 2013 at Fujian Medical University Union Hospital (FMUUH). Patients who underwent emergency surgery, preoperative blood transfusion or neoadjuvant chemotherapy or who had nonprimary adenocarcinoma, T4b tumors, distant metastasis, or gastric remnant carcinoma were excluded from this study. Additionally, patients with no available pretreatment full blood cell count or incomplete clinical and pathological data were also excluded. Clinicopathological data and survival status were recorded. Cancer stage was determined based on the 8th edition of the American Joint Committee on Cancer (AJCC) TNM classification system [23] (Additional file 1: Table S1). Patients with postoperative stage II or higher tumors were recommended to receive adjuvant chemotherapy with 5-FU-based regimens.

### Definition

The laboratory tests were evaluated 1 week before surgery. The white blood cell to hemoglobin ratio (WHR), lymphocyte to monocyte ratio (LMR), neutrophil to lymphocyte ratio (NLR), and platelet to lymphocyte ratio (PLR) were calculated using the following formulas: (white blood cell count [number/mm^3^]) / (10*hemoglobin level [g/L]); (lymphocyte count [number/mm^3^]) / (monocyte count [number/mm^3^]) [12–14]; (neutrophil count [number/mm^3^]) / (lymphocyte count [number/mm^3^]) [6–8]; (platelet count [number/mm^3^]) / (lymphocyte count [number/mm^3^]) [9–11].

### Statistical analysis

The continuous variables are reported as the means ± SD. Youden’s index was calculated using receiver operating characteristic (ROC) analysis to determine an optimal cutoff values for the WHR, NLR, PLR, and LMR for the overall survival analysis. The OS was calculated according to the Kaplan–Meier method. Univariate and multivariate comparisons between groups were performed with log rank tests and cox regression analysis. The C-index was calculated to evaluate the discriminatory ability of the WHR, NLR, LMR, and PLR, and differences between the C-index and area under the curve (AUC) values were examined.

## Results

Table 1 lists the clinical and pathological characteristics of the 924 patients (703 men and 221 women). The average age was 60.9 ± 11.2 years. The means for the WHR, PLR, LMR, and NLR values were 3.2 ± 2.1, 153.7 ± 77.6, 4.4 ± 1.9, and 2.6 ± 2.2, respectively. The pathological TNM stage of the tumor was IA for 195 patients, IB for 72 patients, IIA for 117 patients, IIB for 104 patients, IIIA for 98 patients, IIIB for 159 patients, and IIIC for 179 patients. The median follow-up time was 54 months (IQR, 35–67 months). A total of 62.1% (408/657) of the patients with tumors at postoperative stage II or higher received adjuvant chemotherapy with 5-FU-based regimens.

**Table 1:** Clinical and pathological characteristics

Characteristics	n (%) or Means ± SD
Sex	
Male	703 (76.1%)
Female	221 (23.9%)
Age, yrs.	60.9 ± 11.2
Body mass index (kg/m^2^)	21.9 ± 3.3
ASA scores	
1	335 (38.4%)
2	536 (58%)
3	33 (3.6%)
Comorbidities	
Yes	249 (26.9%)
No	675 (73.1%)
Tumor location	
Upper	221 (23.9%)
Middle	153 (16.6%)
Lower	440 (47.6%)
Mix	110 (11.9%)
WHR	3.2 ± 2.1
PLR	153.7 ± 77.6
LMR	4.4 ± 1.9
NLR	2.6 ± 2.2
Type of surgery	
Total gastrectomy	447 (48.4%)
Partial gastrectomy	477 (51.6%)
Tumor size (cm)	4.6 ± 2.5
Adjuvant chemotherapy	
Yes	408 (44.2%)
No	516 (55.8%)
Pathological T stage	
T1	232 (25.1%)
T2	101 (10.9%)
T3	279 (30.2%)
T4a	312 (33.8%)
Pathological N stage	
N0	359 (38.9%)
N1	134 (14.5%)
N2	143 (15.5%)
N3	288 (31.2%)
Pathological TNM stage	
IA	195 (21.1%)
IB	72 (7.8%)
IIA	117 (12.7%)
IIB	104 (11.3%)
IIIA	98 (10.6%)
IIIB	159 (17.2%)
IIIC	179 (19.4%)

*SD* Standard deviation

*ASA* American Society of Anesthesiologists

*WHR* White blood cell to hemoglobin ratio was calculated using the following formula: (white blood cell count [number/mm^3^]) / (10*hemoglobin level [g/L])

*PLR* The platelet to lymphocyte ratio was defined as the absolute platelet count divided by the absolute lymphocyte count

*LMR* The lymphocyte to monocyte ratio was defined as the absolute lymphocyte count divided by the absolute monocyte count

*NLR* The neutrophil to lymphocyte ratio was defined as the absolute neutrophil count divided by the absolute lymphocyte

**Fig. 1:**
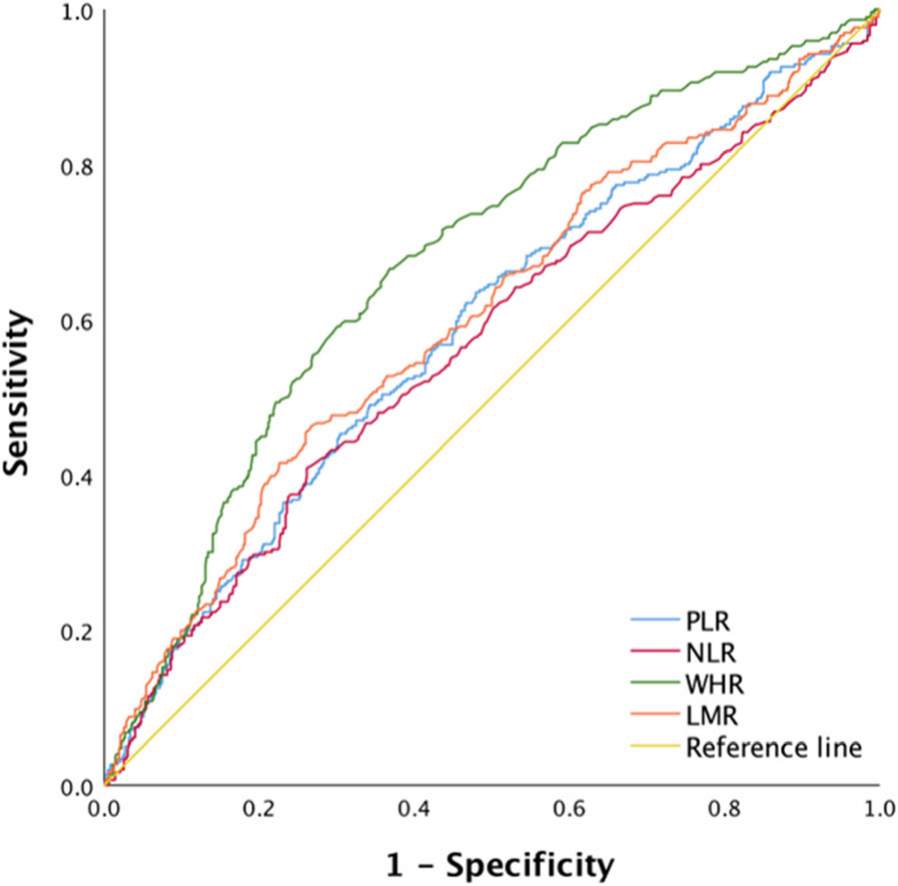
The area under the ROC curve values for WHR (0.675), PLR (5.888), LMR (0.602), and NLR (0.567) as predictors of overall survival

The area under the ROC curve values for WHR (0.675), PLR (5.888), LMR (0.602), and NLR (0.567), which were used to predict 5-year overall survival, are represented in Fig. 1. The optimal cutoff values for the WHR, PLR, LMR, and NLR were 2.855, 133.03, 3.405, and 2.61, respectively, according to Youden’s index, which was calculated using ROC analysis.

**Fig. 2:**
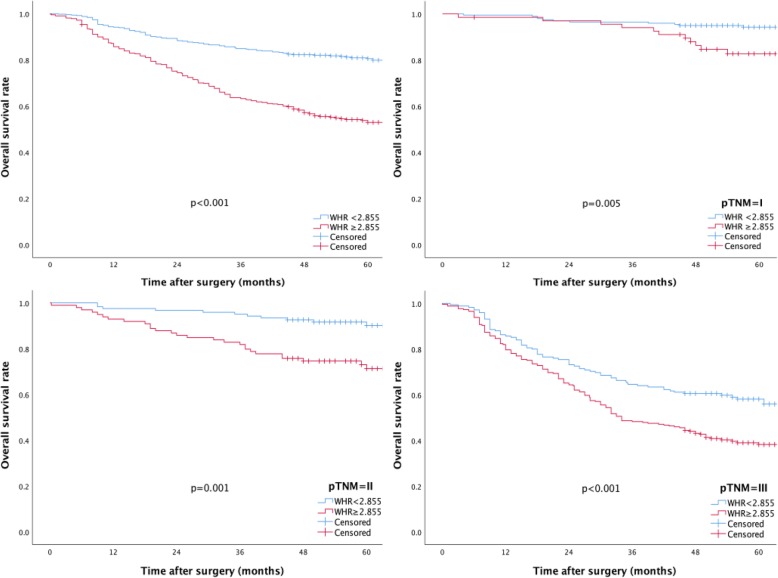
The 5-year OS rates in patients with WHR ≥ 2.855 and WHR < 2.855

The 5-year OS rate in patients with a WHR ≥ 2.855 was significantly lower than that in patients with a WHR < 2.855 (53% vs 88.1%, *p* < 0.001). Further analysis revealed that for patients with stage I (82.7% vs 94.3%, *p* = 0.005), stage II (71.3% vs 90.2%, *p* = 0.001) and stage III tumors (38.2% vs 58.1%, *p* < 0.001), the 5-year OS rates in the WHR ≥ 2.855 group were significantly lower than those in the WHR < 2.855 group (Fig. 2).

Patients with an increased PLR (60.9% vs 75.6%, *p* < 0.001) and NLR (56.7% vs 72.8%, *p* < 0.001) and a decreased LMR (54% vs 74.5%, *p* < 0.001) had a significantly decreased 5-year OS. However, stratified analysis showed that there was no significant 5-year survival rate difference between patients at each stage (Additional file 2: Figures S1-S3).

Multivariate Cox regression analysis demonstrated that the WHR (HR = 8.14, 95% CI 5.14–12.89, *p* < 0.001), PLR (HR = 1.38, 95% CI 1.08–1.75, *p* = 0.009), LMR (HR = 0.62, 95% CI 0.49–0.78, *p* < 0.001) and NLR (HR = 1.33, 95% CI 1.05–1.68, *p* = 0.018) were independent prognostic factors for 5-year OS. After adjusting the WHR, PLR, LMR, and NLR together, only the WHR (HR 1.58, 95% CI 1.2–2.06, p = 0.001) and the LMR (HR 0.71, 95% CI 0.55–0.94, *p* = 0.014) were found to be independent prognostic factors (Table 2).

**Table 2:** Multivariate cox regression analyses demonstrating association of WHR, PLR, LMR and NLR with overall survival

	Variable	Hazard Ratio (95% CI)	*p* value
Model 1^a^	WHR category (≥2.855 vs. < 2.855)	8.14 (5.14–12.89)	< 0.001
Model 2^b^	PLR category (≥133.03 vs. < 133.03)	1.38 (1.08–1.75)	0.009
Model 3^c^	LMR category (≥3.405 vs. < 3.405)	0.62 (0.49–0.78)	< 0.001
Model 4^d^	NLR category (≥2.61 vs. < 2.61)	1.33 (1.05–1.68)	0.018
Model 5^e^	WHR category (≥2.855 vs. < 2.855)	1.58 (1.2–2.06)	0.001
	PLR category (≥133.03 vs. < 133.03)	1.1 (0.85–1.43)	0.477
	LMR category (≥3.405 vs. < 3.405)	0.71 (0.55–0.94)	0.014
	NLR category (≥2.61 vs. < 2.61)	1.03 (0.79–1.36)	0.817

^a^Adjusted for WHR, age, sex, ASA, adjuvant chemotherapy, comorbidity, complication, tumor size, vascular invasion, and cancer stage

^b^Adjusted for PLR, age, sex, ASA, adjuvant chemotherapy, comorbidity, complication, tumor size, vascular invasion, and cancer stage

^c^Adjusted for LMR, age, sex, ASA, adjuvant chemotherapy, comorbidity, complication, tumor size, vascular invasion, and cancer stage

^d^Adjusted for NLR, age, sex, ASA, adjuvant chemotherapy, comorbidity, complication, tumor size, vascular invasion, and cancer stage

^e^Adjusted for WHR, PLR, LMR, NLR, age, sex, ASA, adjuvant chemotherapy, comorbidity, complication, tumor size, vascular invasion, and cancer stage

**Fig. 3:**
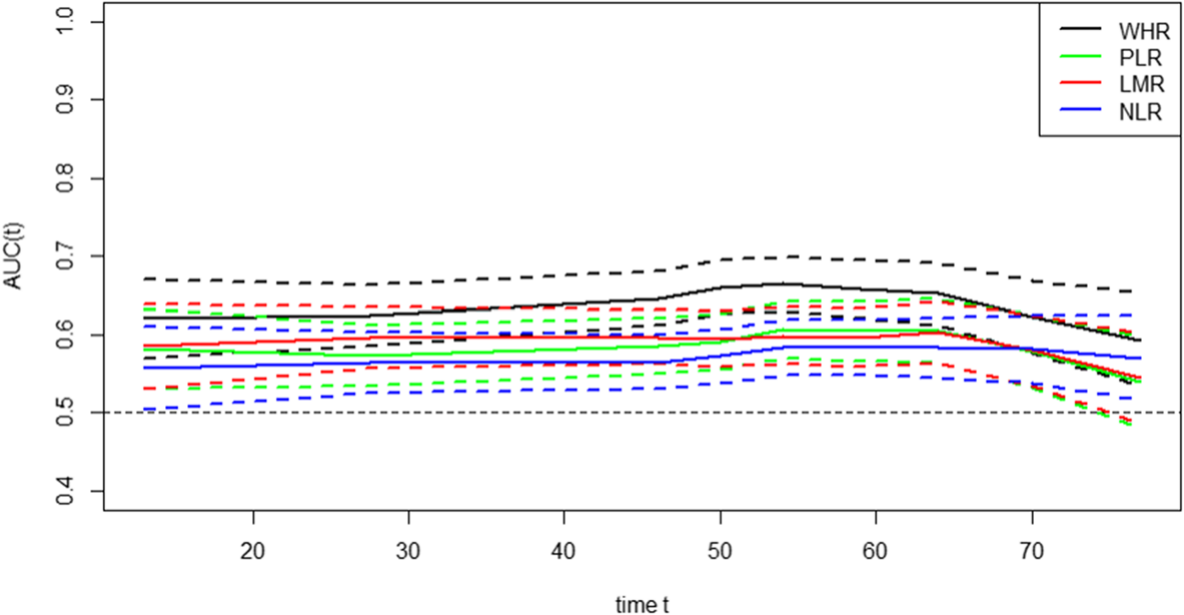
The time-ROC curve comparing the predictive value of the WHR, PLR, LMR, and NLR for the prognosis of gastric cancer. The results showed that the WHR was superior to the PLR, LMR, and NLR during follow-up

By establishing a time-ROC curve to compare the predictive values of the WHR, PLR, LMR, and NLR for the prognosis of gastric cancer (Fig. 3), the results showed that the WHR was superior to the PLR, LMR, and NLR in terms of predictive value during follow-up. The WHR C-index (0.624) was significantly greater than the PLR (0.569), LMR (0.584), and NLR C-indexes (0.56) (all *P* < 0.001, Table 3). Patients were divided into a high-risk group and a low-risk group according to the optimal threshold of the inflammatory index. The analysis showed that the WHR still distinguished each subgroup determined according to the PLR, LMR, and NLR (Additional file 2: Figures S4-S7). In addition, the WHR had good predictive value for various clinical subgroups, such as those determined according age, sex, BMI, tumor location and tumor size (Additional file 2: Figure S8).

**Table 3:** Comparison of the C-index between the inflammations

Variables	C-index	95% CI	*p* value
WHR	0.624	0.597–0.652	
PLR	0.569	0.54–0.597	< 0.001
LMR	0.584	0.566–0.612	< 0.001
NLR	0.56	0.532–0.588	< 0.001

Comparison of the C-index between the WHR and other inflammations was performed using the Z test method

## Discussion

This report assessed the value of preoperative inflammation-based prognostic scores (PIPS), which are derived from a full blood cell count, in predicting the overall survival (OS) rate in patients with resectable gastric cancer. We found that the WHR (HR 8.14, 95% CI 5.14–12.89, *p* < 0.001) was an independent predictor of overall survival and had a significantly better capability to predict overall survival than the PLR, LMR, and NLR in gastric cancer. Furthermore, the WHR could not only discriminate all patients well but could also discriminate subgroup patients who were divided into high-risk and low-risk groups according to the optimal cutoff values for PLR, LMR, and NLR.

Various preoperative inflammation-based prognostic scores (PIPS), which are derived from a full blood cell count, were found to be strong predictors in patients with malignant disease. PLR has been shown to be associated with decreased survival in patients with several malignant tumors, such as colorectal cancer [24], pancreatic ductal adenocarcinoma [25], and gastric cancer [26]. Eva Lieto et al. reported that LMR was linked to prognosis and could be used to easily predict outcomes in gastric cancer patients undergoing surgery [13, 14, 27]. Jiang N et al. demonstrated that the NLR represents a useful prognostic factor for the prediction of overall survival (OS) in patients with advanced gastric cancer [26], and Mohri Y et al. showed that a high pretreatment NLR was an independent prognostic factor in patients with metastatic gastric cancer [28]. In our study, the PIPS cutoff value was determined based on the ROC curve rather than cutoff values already determined in other published studies. The study also revealed that the PLR, LMR, and NLR were independent prognostic factors; however, after adjusting for the WHR, PLR, LMR, and NLR simultaneously, only the WHR (HR 1.58, 95% CI 1.2–2.06, *p* = 0.001) and LMR (HR 0.71, 95% CI 0.55–0.94, *p* = 0.014) were found to be independent prognostic factors. Further analysis showed that patients with a WHR ≥ 2.855 and postoperative stage III tumors who were treated with chemotherapy had significantly improved 5-year overall survival; however, patients with a WHR < 2.855 who were treated with chemotherapy did not show an improvement in 5-year overall survival (Additional file 2: Figures S9-S10).

The underlying mechanisms related to PIPS in patients with malignant disease are still unclear. Nash GF et al. demonstrated that PLTs are unarguably associated with the growth and spread of cancer and hypothesized that the mechanism involved in the inhibition by thrombocytopenia of the spread of cancer was through fibronectin and von Willebrand factor, which bridged the platelet–integrin–tumor interaction [29]. Hoffmann et al. believed that low lymphocyte counts led to decreased survival because of an insufficient immunological reaction [30, 31]. Furthermore, a study by L. Zheng confirmed that the PLR and NLR were significantly associated with increases in the circulating tumor cell (CTC) count and the circulating tumor cell count detection ratio [32]. These correlations suggested that the measurement of inflammatory markers may enhance the detection of CTCs in individual patients with gastric cancer. Tumor-associated macrophages (TAMs), which are differentiated from monocytes, are also involved in oncogenesis. TAMs can lead to tumor growth, invasion, migration and recidivism through accelerating angiogenesis [33, 34]. Based on this, we hypothesized that the PLR, LMR, and NLR reflect the balance between host immune status and tumor progression and thus have the capability to predict the prognosis of patients with gastric cancer.

WBCs are considered one of the most important components of the immune system and are associated with protecting the body from both foreign invaders and infectious disease. It is not enough to evaluate the immune system just by measuring specific blood cells, and we believe that, because they are the most important components of the immune system, WBCs should be considered an integral cell type. The presence of pretreatment anemia has been reported to be associated with poor prognosis in many types of malignancies [35–38]. Xuechao Liu et al. found that patients with TNM stage III gastric cancer had decreased survival if they had mild preoperative anemia [38]. There is evidence that low hemoglobin levels can lead to poor tumor oxygenation [39], and clinical studies revealed that hypoxia was an independent risk factor for poor prognosis in patients with malignancies [40–43]. A hypoxic microenvironment not only can directly increase tumor cell invasiveness and accelerate metastasis but can also cause resistance to chemotherapy and radiotherapy via mechanisms involved in proteomic and genomic changes. Based on these findings, we hypothesize that the WHR may reflect the balance between host immune status and tumor progression and predict the prognosis of patients with gastric adenocarcinoma.

There are some limitations of this study. It is a retrospective analysis and is therefore subject to the typical bias associated with this type of data collection. In addition, there was no evaluation of other factors, such as the lymph node rate, mGPS and histological subtype, that are also associated with outcomes.

The value of full blood cell count-based preoperative inflammation prognostic scores for the prediction of prognosis in patients with gastric cancer needs to be clarified in further large-scale prospective studies. In particular, the reason why WHR has the most power to stratify and predict the long-term outcomes of patients with gastric adenocarcinoma compared with other PIPS remains to be elucidated.

## Conclusion

This report assessed the value of PIPS in predicting the overall survival (OS) rate in patients with resectable gastric adenocarcinoma. To the best of our knowledge, this is the first study to investigate the value of the white blood cell to hemoglobin ratio (WHR). We found that compared with other PIPS, the WHR had the most powerful capability to predict long-term outcomes in patients with gastric adenocarcinoma.

## Supplementary information

**Additional file 1: Table S1.** A comparison of the 7th and 8th editions of the AJCC staging system.

**Additional file 2: Figure S1.** The 5-year OS rates in patients with PLR ≥ 133.03 and PLR < 133.03. Patients with an increased PLR (60.9% vs 75.6%, *p* < 0.001) had significantly decreased 5-year OS. However, the stratified analysis showed that the 5-year OS rates in patients with stage I (91.5% vs 91.4%, *p* = 0.995) and stage II tumors (79.1% vs 84.6%, *p* = 0.228) showed no significant differences, whereas the 5-year OS rates in patients with stage III tumors (39.3% vs 56.1%, *p* = 0.004) showed significant differences. **Figure S2.** The 5-year OS rates in patients with LMR ≥ 3.405 and LMR < 3.405. Patients with a decreased LMR (54% vs 74.5%, *p* < 0.001) showed significantly deceased 5-year OS. However, stratified analysis showed that the 5-year OS rates in patients with stage I (89.1% vs 92%, *p* = 0.446) and stage II tumors (72.6% vs 85.9%, *p* = 0.052) showed no significant differences, whereas the 5-year OS rates in patients with stage III tumors (34.6% vs 53.9%, *p* < 0.001) showed significant differences. **Figure S3.** The 5-year OS rates in patients with NLR ≥ 2.61 and NLR < 2.61. Patients with an increased NLR (56.7% vs 72.8%, *p* < 0.001) had a significantly decreased 5-year OS. However, the stratified analysis showed that the 5-year OS rates in patients with stage I tumors (91.5% vs 91.4%, *p* = 0.953) showed no significant differences, whereas the 5-year OS rates in patients with stage II (71.4% vs 85.4%, *p* = 0.013) and stage III tumors (38.6% vs 50.9%, *p* = 0.043) showed significant differences. **Figure S4.** The WHR distinguished each subgroup determined according to the PLR, LMR, and NLR. Patients were divided into a high-risk group and a low-risk group according to the optimal threshold of the inflammatory index. The analysis showed that the WHR still distinguished each subgroup determined according to the PLR, LMR, and NLR. **Figure S5.** The PLR distinguished each subgroup determined according to the WHR, LMR, and NLR. Patients were divided into a high-risk group and a low-risk group according to the optimal threshold of the inflammatory index. The analysis showed that the PLR could not distinguish each subgroup determined according to the WHR, LMR, and NLR. **Figure S6.** The LMR distinguished each subgroup determined according to the WHR, PLR and NLR. Patients were divided into a high-risk group and a low-risk group according to the optimal threshold of the inflammatory index. The analysis showed that the LMR could not distinguish each subgroup determined according to the WHR, PLR and NLR. **Figure S7.** The NLR distinguished each subgroup determined according to the WHR, PLR and LMR. Patients were divided into a high-risk group and a low-risk group according to the optimal threshold of the inflammatory index. The analysis showed that the NLR could not distinguish each subgroup determined according to the WHR, PLR and LMR. **Figure S8.** The WHR has good predictive value for the various clinical subgroups. WHR had good predictive value for the various clinical subgroups, such as those determined according to age, sex, BMI, tumor location and tumor size. **Figure S9.** The therapeutic effect of chemotherapy in patients with a WHR < 2.855. Patients with a WHR ≥ 2.855 and postoperative stage III tumors who were treated with chemotherapy had significantly improved 5-year overall survival. **Figure S10.** The therapeutic effect of chemotherapy in patients with a WHR ≥ 2.855. Patients with a WHR < 2.855 who were treated with chemotherapy did not show an improvement in 5-year overall survival.

## Data Availability

The dataset analyzed in this study is available from the corresponding author upon reasonable request.

## Abbreviations

ASA: American Society of Anesthesiologists
AUC: Area under the curve
BMI: Body mass index
CI: Confidence interval
LMR: Lymphocyte to monocyte ratio
NLR: Neutrophil to lymphocyte ratio
OS: Overall survival
PIPS: Preoperative inflammation-based prognostic scores
PLR: Platelet to lymphocyte ratio
ROC: Receiver operating characteristic
SD: Standard deviation
WHR: White blood cell to hemoglobin ratio
