# Supplementary material for: Exploring the value of new preoperative inflammation prognostic score: white blood cell to hemoglobin for gastric adenocarcinoma patients
Source: BMC Cancer. 2019 Nov 21;19:1127. doi: 10.1186/s12885-019-6213-0 (PMC6868868; doi:10.1186/s12885-019-6213-0)
Supplement: Supplementary file 1 — Additional file 1: Table S1. A comparison of the 7th and 8th editions of the AJCC staging system. [file 12885_2019_6213_MOESM1_ESM.docx]

Additional file 1: Table S1 A comparison of the 7th and 8th editions of the AJCC staging system

| AJCC seventh edition | N0 | N1(1-2) | N2(3-6) | N3(7-) | Any N, M1 |
| --- | --- | --- | --- | --- | --- |
| T1 | IA | IB | IIA | IIB | IV |
| T2 | IB | IIA | IIB | IIIA |  |
| T3 | IIA | IIB | IIIA | IIIB |  |
| T4a | IIB | IIIA | IIIB | IIIC |  |
| T4b | IIIB | IIIB | IIIC | IIIC |  |
| Any T, M1 |  | | | | |

| AJCC eighth edition | N0 | N1(1-2) | N2(3-6) | N3a(7-15) | N3b(16-) | Any N, M1 |
| --- | --- | --- | --- | --- | --- | --- |
| T1 | IA | IB | IIA | IIB | IIIB | IV |
| T2 | IB | IIA | IIB | IIIA | IIIB |  |
| T3 | IIA | IIB | IIIA | IIIB | IIIC |  |
| T4a | IIB | IIIA | IIIA | IIIB | IIIC |  |
| T4b | IIIA | IIIB | IIIB | IIIC | IIIC |  |
| Any T, M1 |  | | | | | |
