# Supplementary material for: Exploring the value of new preoperative inflammation prognostic score: white blood cell to hemoglobin for gastric adenocarcinoma patients
Source: BMC Cancer. 2019 Nov 21;19:1127. doi: 10.1186/s12885-019-6213-0 (PMC6868868; doi:10.1186/s12885-019-6213-0)
Supplement: Supplementary file 2 — Additional file 2: Figure S1. The 5-year OS rates in patients with PLR ≥ 133.03 and PLR < 133.03. Patients with an increased PLR (60.9% vs 75.6%, p < 0.001) had significantly decreased 5-year OS. However, the stratified analysis showed that the 5-year OS rates in patients with stage I (91.5% vs 91.4%, p = 0.995) and stage II tumors (79.1% vs 84.6%, p = 0.228) showed no significant differences, whereas the 5-year OS rates in patients with stage III tumors (39.3% vs 56.1%, p = 0.004) showed significant differences. Figure S2. The 5-year OS rates in patients with LMR ≥ 3.405 and LMR < 3.405. Patients with a decreased LMR (54% vs 74.5%, p < 0.001) showed significantly deceased 5-year OS. However, stratified analysis showed that the 5-year OS rates in patients with stage I (89.1% vs 92%, p = 0.446) and stage II tumors (72.6% vs 85.9%, p = 0.052) showed no significant differences, whereas the 5-year OS rates in patients with stage III tumors (34.6% vs 53.9%, p < 0.001) showed significant differences. Figure S3. The 5-year OS rates in patients with NLR ≥ 2.61 and NLR < 2.61. Patients with an increased NLR (56.7% vs 72.8%, p < 0.001) had a significantly decreased 5-year OS. However, the stratified analysis showed that the 5-year OS rates in patients with stage I tumors (91.5% vs 91.4%, p = 0.953) showed no significant differences, whereas the 5-year OS rates in patients with stage II (71.4% vs 85.4%, p = 0.013) and stage III tumors (38.6% vs 50.9%, p = 0.043) showed significant differences. Figure S4. The WHR distinguished each subgroup determined according to the PLR, LMR, and NLR. Patients were divided into a high-risk group and a low-risk group according to the optimal threshold of the inflammatory index. The analysis showed that the WHR still distinguished each subgroup determined according to the PLR, LMR, and NLR. Figure S5. The PLR distinguished each subgroup determined according to the WHR, LMR, and NLR. Patients were divided into a high-risk group and a low-risk gr [file 12885_2019_6213_MOESM2_ESM.pptx]

## Slide 1
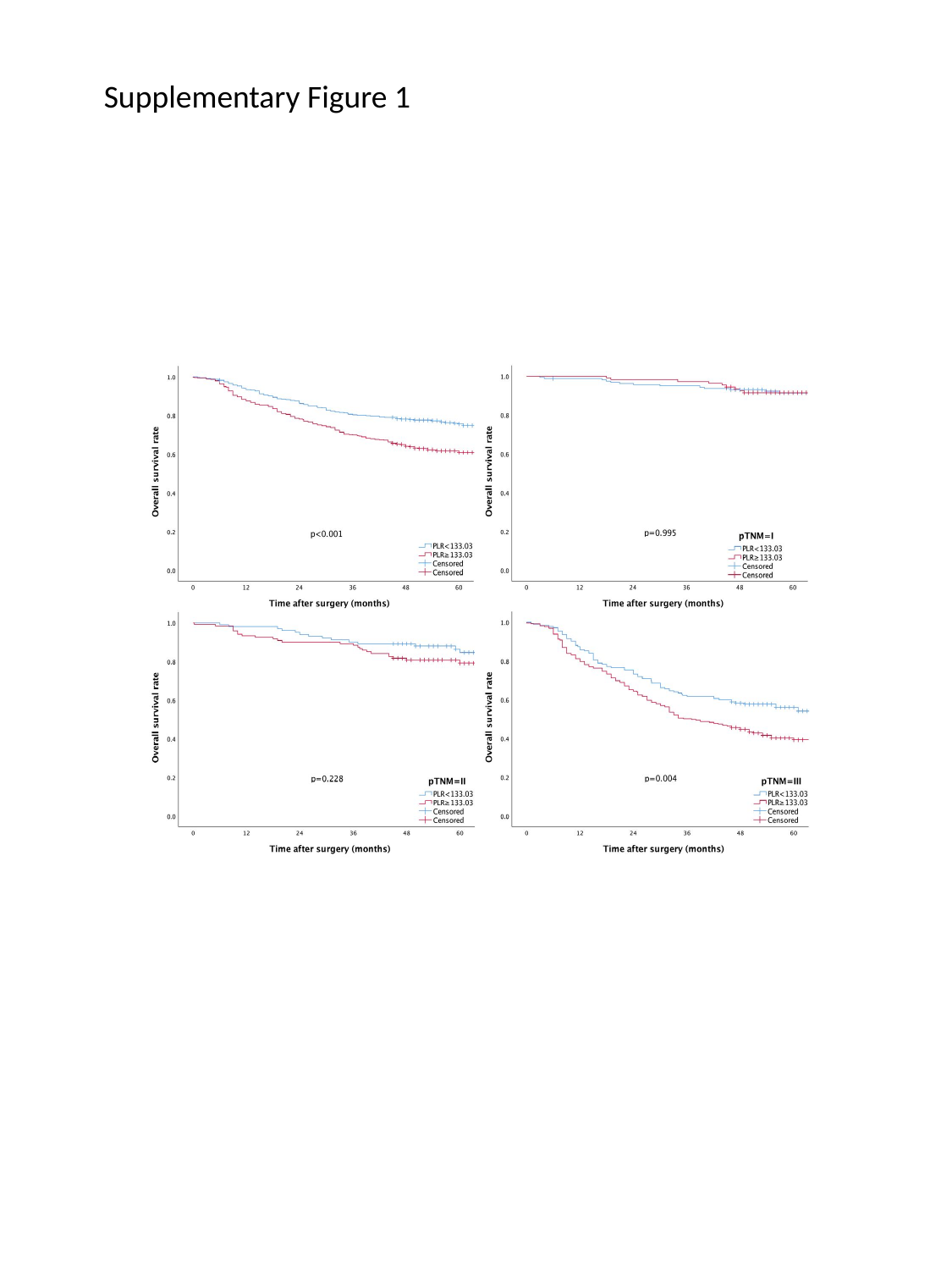

Supplementary Figure 1

## Slide 2
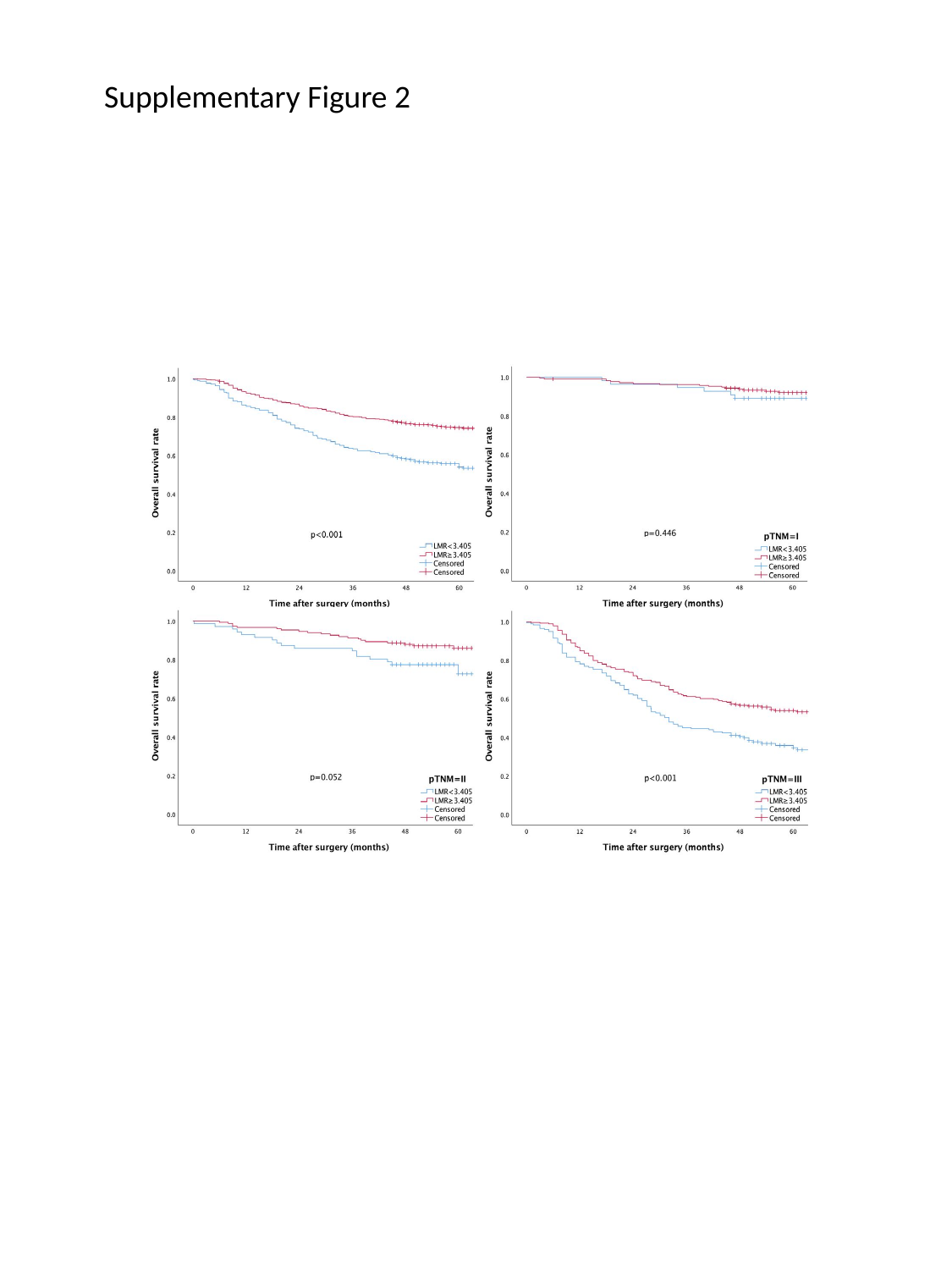

Supplementary Figure 2

## Slide 3
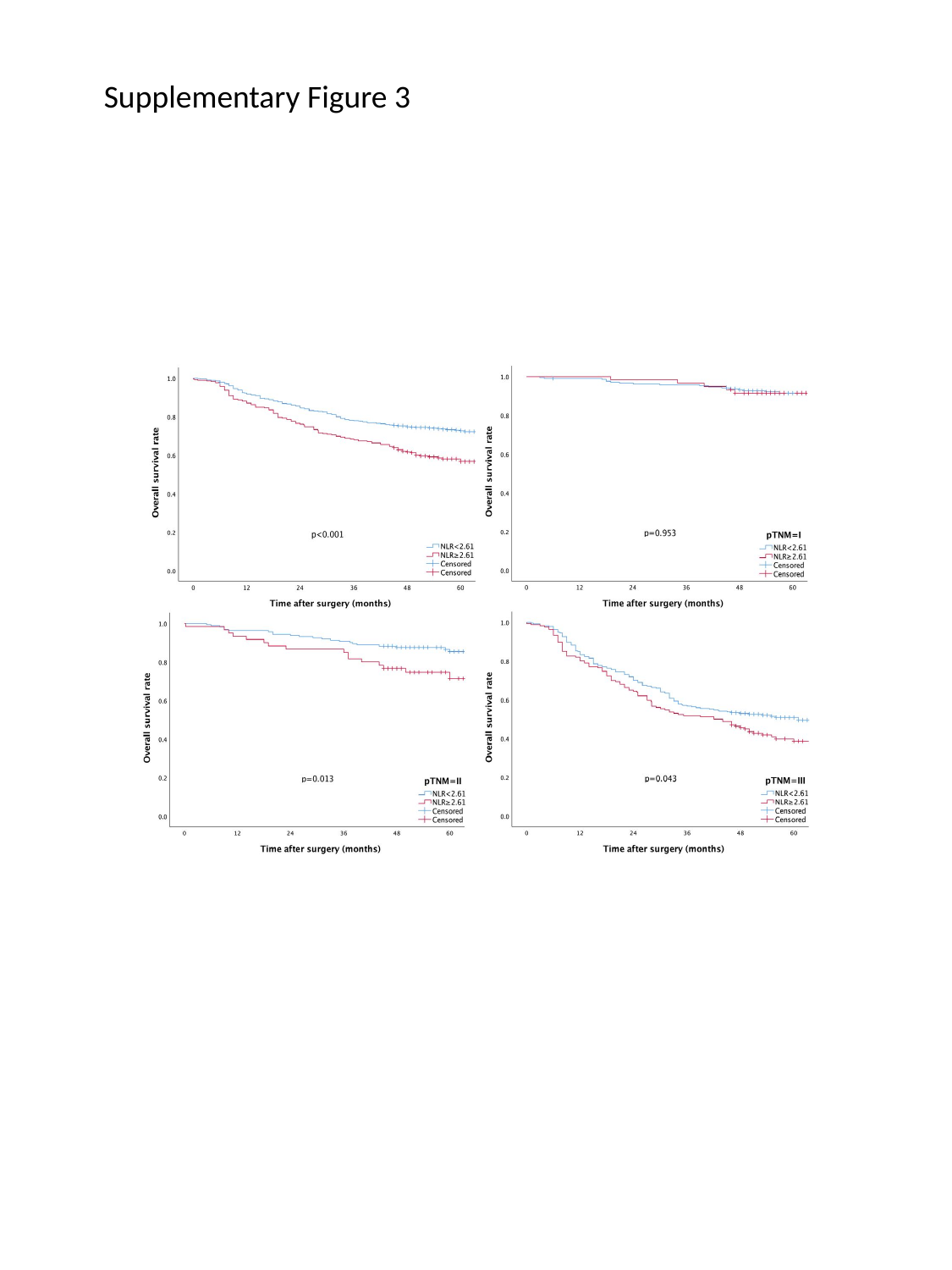

Supplementary Figure 3

## Slide 4
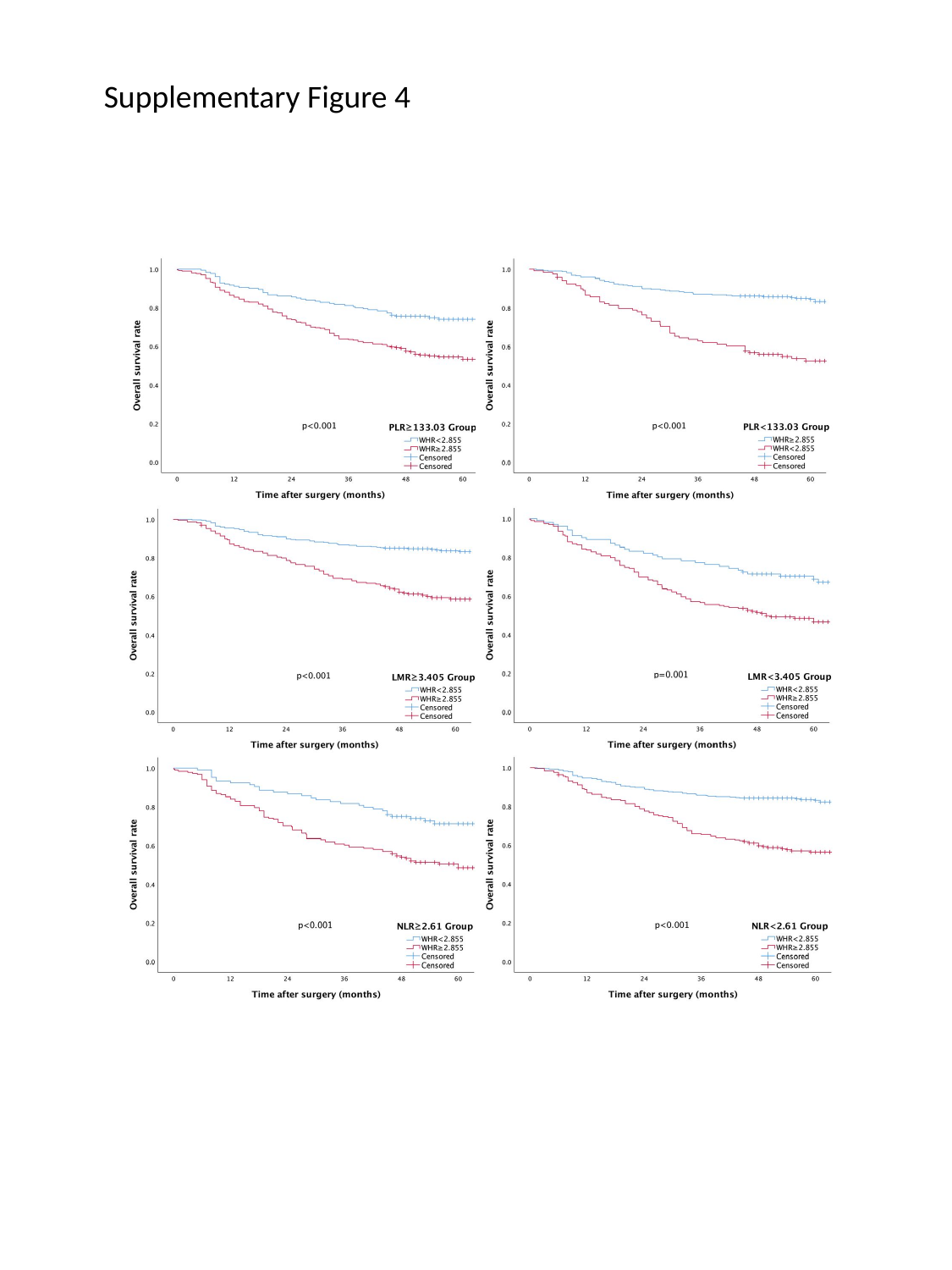

Supplementary Figure 4

## Slide 5
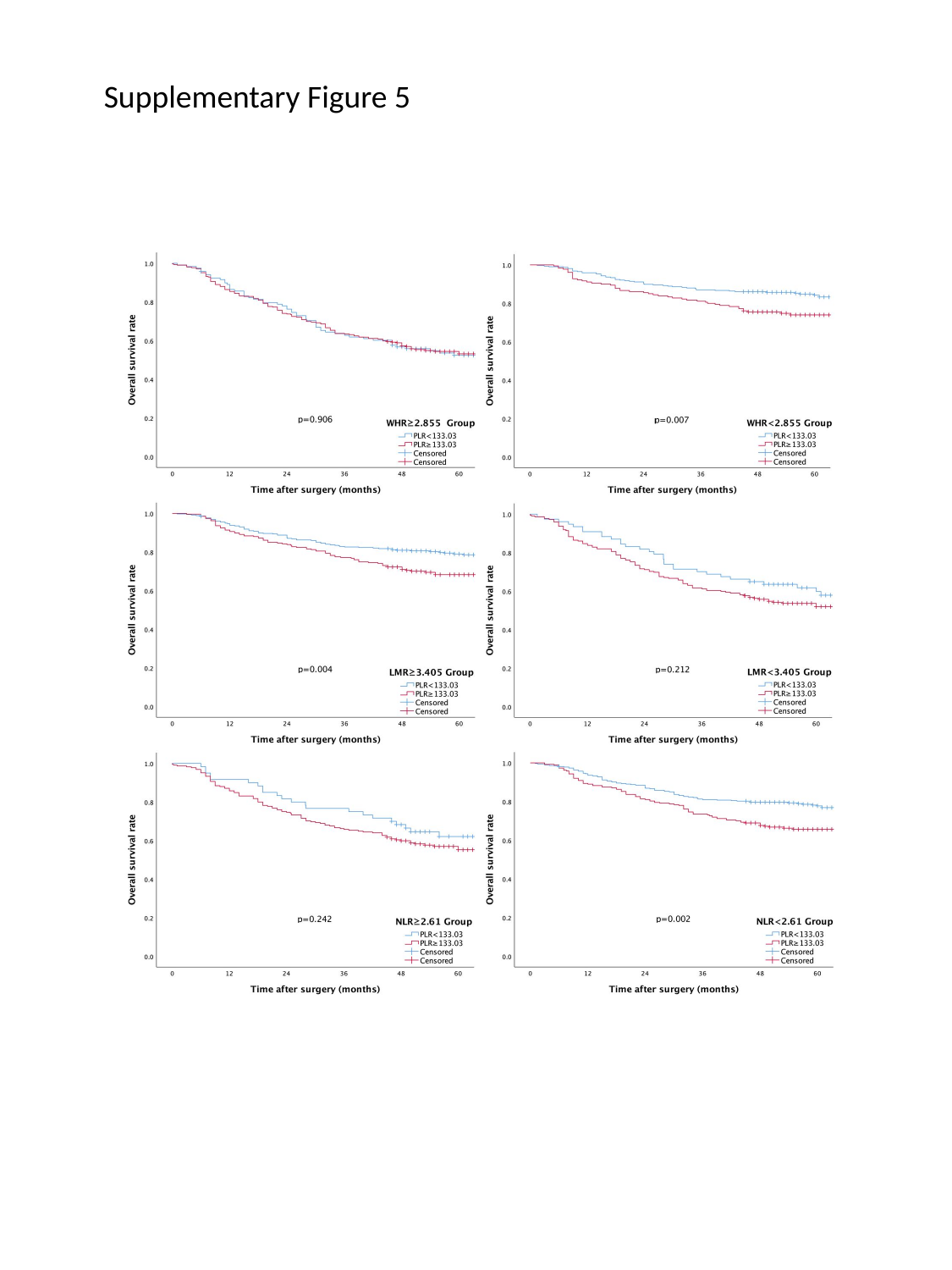

Supplementary Figure 5

## Slide 6
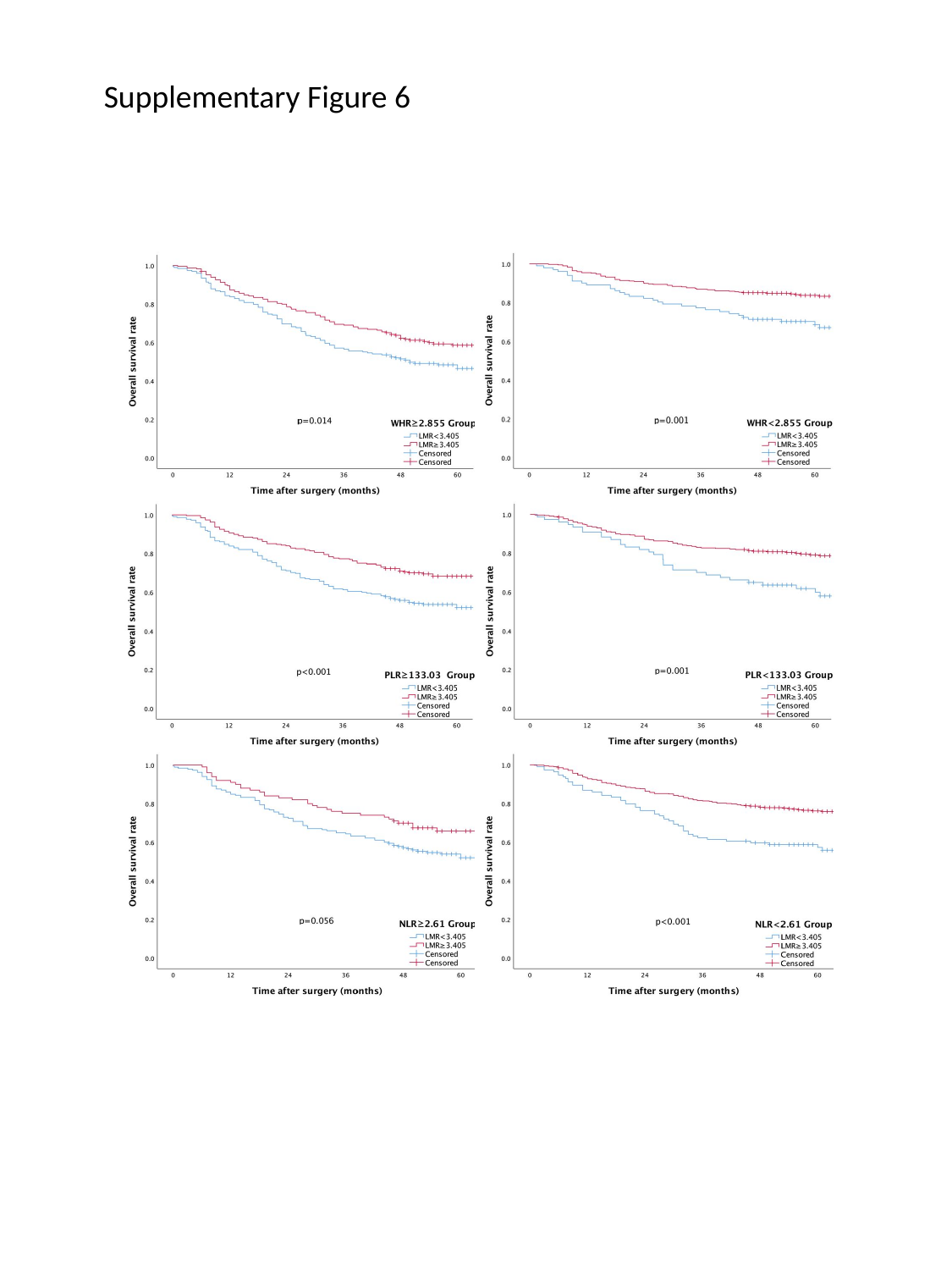

Supplementary Figure 6

## Slide 7
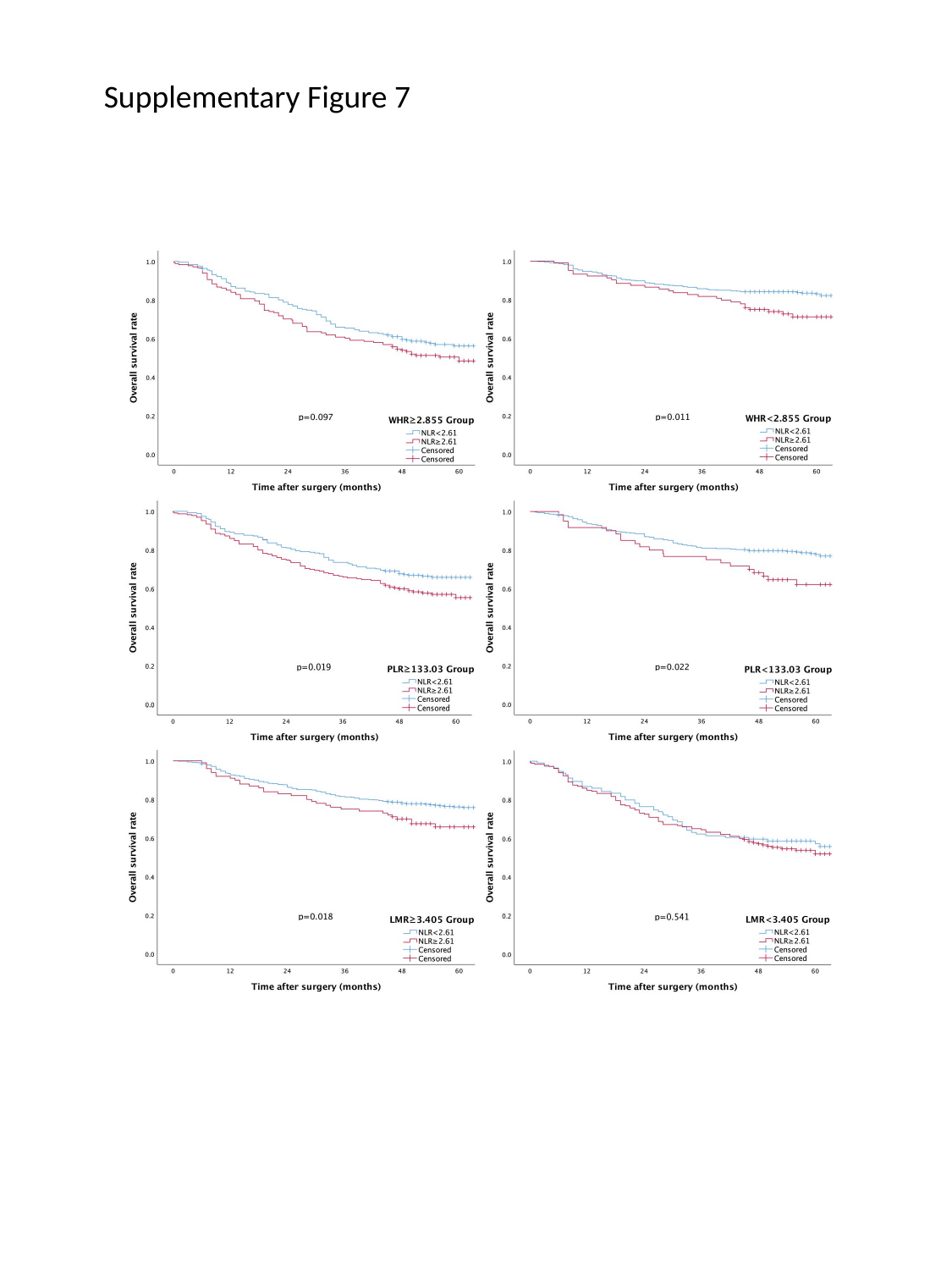

Supplementary Figure 7

## Slide 8
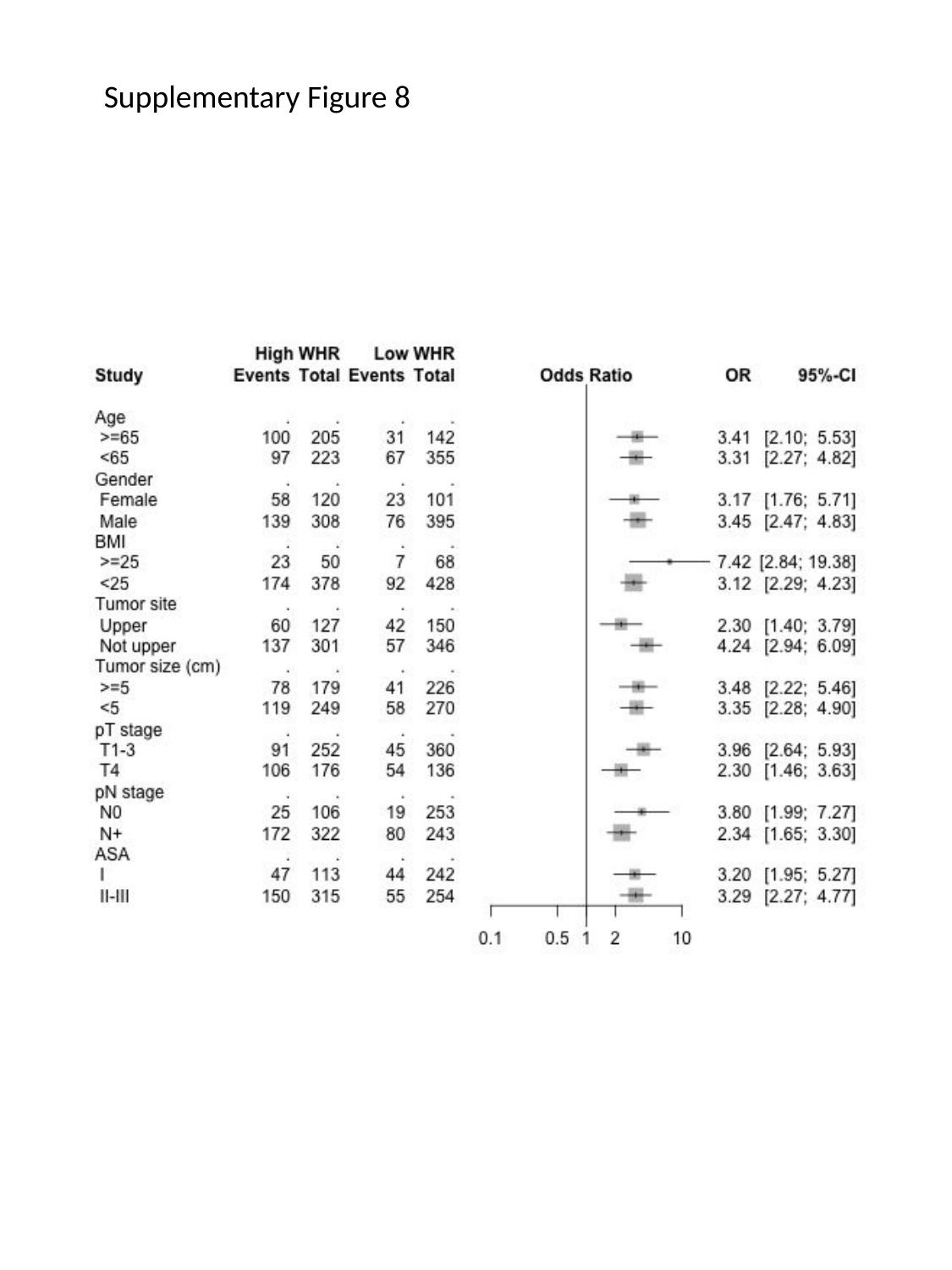

Supplementary Figure 8

## Slide 9
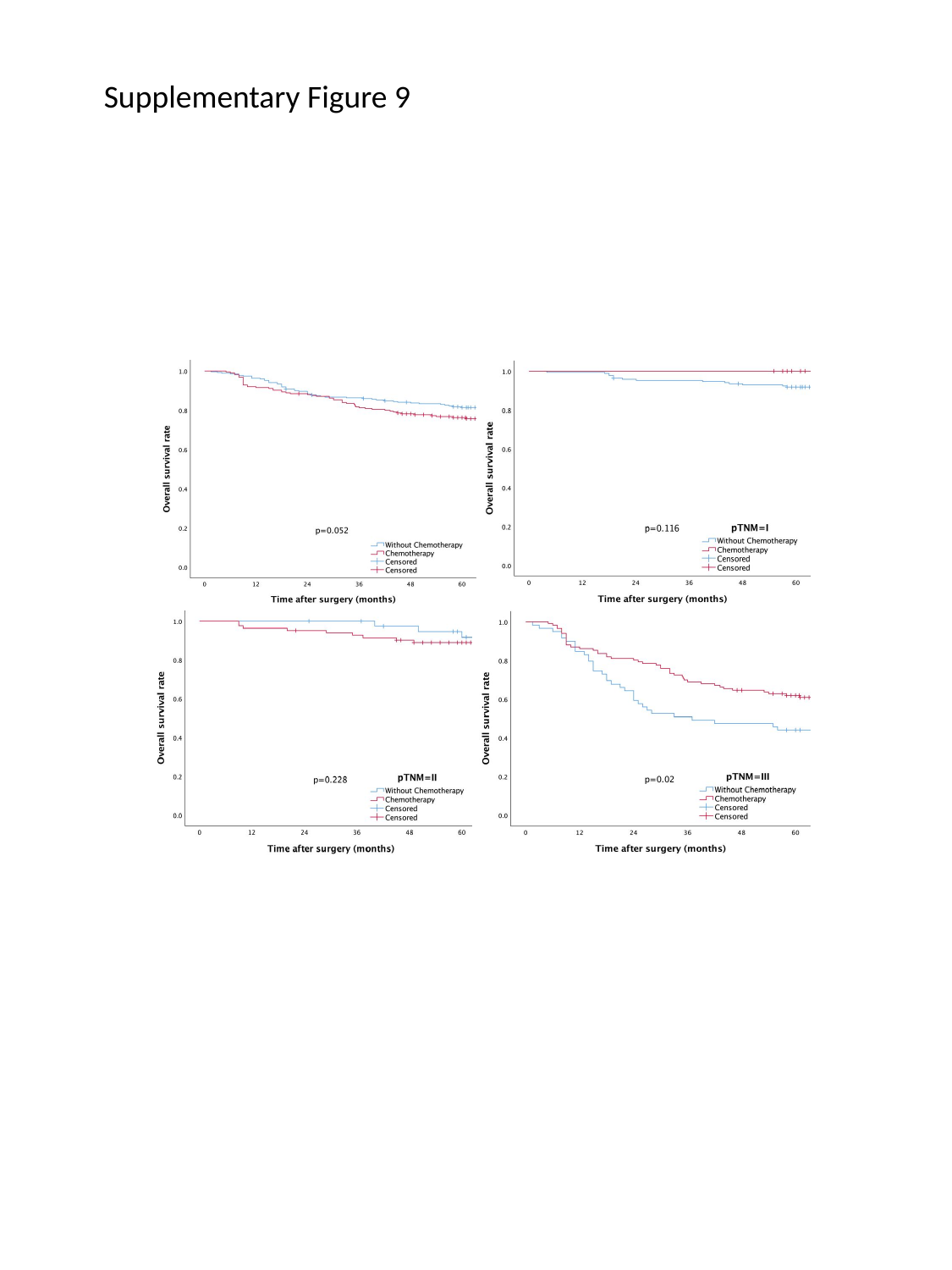

Supplementary Figure 9

## Slide 10
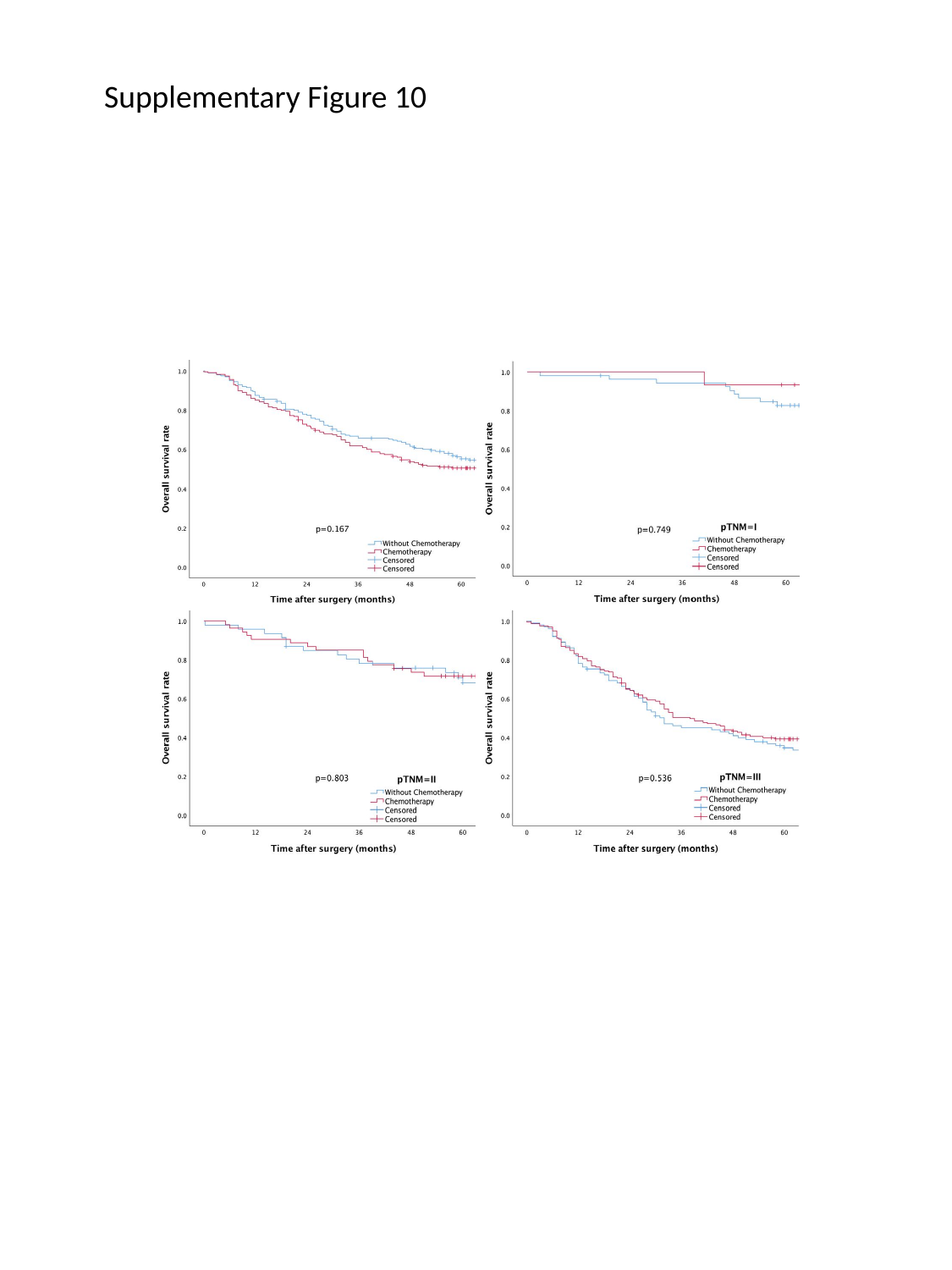

Supplementary Figure 10
